# Maternal Pressure and Frequent Use of Bottle During Feeding Moderate Infant Food Cue Reactivity over Time

**DOI:** 10.3390/nu17223605

**Published:** 2025-11-18

**Authors:** Lenka H. Shriver, Yu Chen, Cheryl Buehler, Laurie Wideman, Esther M. Leerkes

**Affiliations:** 1 Department of Nutrition, University of North Carolina at Greensboro, P.O. Box 26170, Greensboro, NC 27402-6170, USA; 2 Department of Human Development and Family Studies, University of North Carolina at Greensboro, P.O. Box 26170, Greensboro, NC 27402-6170, USA; y_chen12@uncg.edu (Y.C.); emleerke@uncg.edu (E.M.L.); 3 Department of Kinesiology, University of North Carolina at Greensboro, P.O. Box 26170, Greensboro, NC 27402-6170, USA; l_widema@uncg.edu

**Keywords:** food cue reactivity, infancy, pressure, feeding style, bottle feeding

## Abstract

**Background**: Food cue reactivity (FCR) has been associated with a higher obesity risk, but little is known about what factors influence FCR during infancy. This study examined the moderating effects of maternal feeding styles and bottle feeding on the associations between 2-month and 6-month FCR. **Methods**: Data came from 299 mother–infant dyads who participated in a larger early-obesity-risk study and provided information via online questionnaires (i.e., socio-demographics) and/or during lab visits (i.e., anthropometrics) prenatally and at 2 and 6 months postpartum. Food-related practices (i.e., bottle use, milk type), feeding styles and FCR were measured by previously validated mother-reported measures: Infant Feeding Practices, Infant Feeding Styles, and Baby Eating Behavior Questionnaires. A regression model with moderation effects between 2-month FCR, feeding styles, and bottle feeding on 6-month FCR was tested using Mplus, controlling for selected variables (i.e., milk type, infant birthweight). Maternal pressure interacted with 2-month FCR and bottle feeding to predict 6-month FCR. **Results**: The moderating role of higher pressure was significant only for infants who received most of their feeds via a bottle (“high” bottle feeding). No other interactions were significant. Maternal feeding pressure in combination with frequent bottle feeding further strengthens the positive association between early and late FCR in infancy. **Conclusions**: Given the previously established links between FCR and weight outcomes among children, reducing the controlling maternal feeding practice of pressure during feeding in infancy, especially among mothers who frequently bottle feed, might be an important intervention target for optimizing weight outcomes in the first year of life.

## 1. Introduction

Nearly 42 million children under age 5 are obese, representing a global public health issue where multiple complex factors interact leading to an imbalance between energy intake and expenditure [1,2]. Food intake is stimulated by physiological responses to hunger and satiety (i.e., homeostatic eating), but it is influenced by biopsychosocial and environmental factors that are unique to each individual, including appetite [3,4]. A better understanding of how appetitive characteristics interact with the child’s care environment early in life is warranted to design more effective prevention strategies [4,5].

Appetitive traits have been found to differ among infants as early as at birth and are believed to be influenced by the child’s environment [6,7]. This is consistent with the Behavioral Susceptibility Theory (BST), which argues that individuals’ genetic predispositions interact with their unique food-related environments to influence weight outcomes [8,9]. According to BST, differences in appetitive traits help shape why some children might overeat while others do not whenever presented with the opportunity. More specifically, this theory suggests that children who are genetically programmed to have greater food approach traits and lower satiety responsiveness are more likely to overeat, especially when exposed to food-rich environments. In other words, their appetitive traits function as a mediator of the link between their genetic predispositions and the food environment they live in [9]. Consequently, some children may be less vulnerable than others to becoming overweight or obese even when living in an obesogenic food environment [9,10,11].

Previous studies show that “food cue reactivity or responsivity” (FCR), defined as “cognitive, emotional, and/or physiological changes that result from exposure to food cues”, is higher among obese children compared to children at a healthy weight [12,13]. Children who have high FCR tend to have a stronger response, either behavioral, psychological and/or physiological, to cues that signal food compared to other children [14]. Children with greater FCR are often characterized by having a big appetite, enjoying food more than others, and/or being more vulnerable to eating even when not hungry when exposed to palatable foods [11,14]. Previous research has linked higher levels of such food approach traits [14] to negative weight-related outcomes across age groups. A meta-analysis of 3292 participants found that FCR and craving explained a significant proportion of the variance in increased food intake and/or weight gain in both adults and children [15]. A systematic review by Kininmonth et al. [16] found that food approach traits were associated with adiposity measures in all 72 studies that were reviewed, including those with children [16]. Importantly, food responsiveness appears to predict rapid infant weight gain, which is strongly associated with a higher obesity risk later in life [17,18,19]. Because infants interact closely with their parents and quickly learn new behaviors, the first few months of life might represent a critical window for obesity-prevention interventions guided by the BST [5,6,9]. In the current literature, however, there is still little understanding of appetitive traits, their correlates, and predictors of FCR trajectories during infancy [16].

Maternal food-related practices play a key role in infants’ food environment, influencing many outcomes [20,21]. Research has identified food-related practices that are associated with a greater obesity risk, including pressure and bottle feeding [22,23]. Pressure to eat is considered one of the “obesogenic” feeding practices because it overrides the infant’s satiation and inhibits the ability to self-regulate food intake, leading to over-eating and a higher obesity risk later in life [20,21,24,25,26]. Controlling maternal feeding style was also found to significantly interact with infant food responsiveness to increase the odds of rapid infant weight gain by 6 months of age [19]. Similarly, bottle feeding, irregardless of milk type, has been implicated as an obesogenic feeding-related practice [23,27], with the frequent use of bottle feeding during the first 6 months of life being associated with higher obesity risk at age 6.

The effectiveness of child obesity treatment programs has been limited, and experts agree that efforts must focus on early life prevention [5]. Thus, it is critical to better understand how appetite traits interact with maternal food-related practices in the first few months of life [16]. This study examined interactions between FCR in early infancy, maternal feeding styles, and bottle feeding on infant FCR at 6 months of age. We hypothesized that greater FCR at 2 months would predict higher FCR later in infancy, but that maternal pressure and/or bottle feeding would interact with early FCR to predict FCR at 6 months. Specifically, we expected that maternal pressure during feeding would exacerbate the association between early and later FCR in our sample. Hypotheses related to the other feeding styles were not proposed, given the lack of research on appetitive traits among infants and the exploratory nature of this study.

## 2. Materials and Methods

### 2.1. Study Design, Participants, and Procedures

Data for this study came from mother–infant dyads participating in a large interdisciplinary early-childhood-obesity risk study in the southeastern urban part of the U.S. Participants were recruited during their 3rd trimester of pregnancy using personal contact and/or advertisement in childbirth and/or prenatal breastfeeding classes, OB/GYN clinics, public health departments, stores/events, and other locations. Social media and other online outlets were also utilized for recruitment (e.g., Facebook, listservs). Flyers and advertisements were posted in physical locations as well as online outlets with QR codes that allowed interested individuals to contact the research staff with questions. The eligibility criteria for participation in the overall study included the following: (1) 18 or older; (2) expecting a singleton; (3) fluent in English; (4) planning to remain in the region for 3 years. Exclusion criteria for the overall study included birth defects, infants born <32 weeks of gestation, and metabolic disorders. The same criteria were utilized for the participants whose data were included in the current study. Once each participant was screened for eligibility, they were asked to review and sign a written informed consent form, and their first lab visit was scheduled to occur during the third trimester of their pregnancy. For the current study, data from the prenatal, 2 m, and 6 m data collection points were utilized. Anthropometric measurements were completed during the lab visits, while other information, such as socio-demographics, feeding-related information, and appetite-related characteristics, were collected via questionnaires using Qualtrics prior to each lab visit. Additional details related to the key variables in the current study are provided below. A total of 299 eligible mother–infant dyads chose to participate and provided at least some information across data collection time points that occurred between 2020 and 2024 (prenatal during 3rd trimester, and then at 2 m, 6 m, 12 m, and 24 m). Further details about the overall study can be found elsewhere [28]. The current study utilized data collected during the prenatal, 2-, and 6-month timepoints.

### 2.2. Study Variables and Measures

#### 2.2.1. Food Cue Reactivity

Food cue reactivity (FCR) was operationalized as a sum of three food approach subscales from the Baby Eating Behavior Questionnaire (BEBQ), a parent-reported measure, that was validated in previous research [7,29]. The following subscales were utilized as continuous variables to create the FCR score at both timepoints: Food Responsiveness (6 items; “My baby is always demanding food”), Enjoyment of Food (4 items; “My baby enjoys feeding time”), and General Appetite (1 item; “My baby has a big appetite”). The responses for each subscale ranged from 1—never to 5—always. Internal consistency of FCR was good at both 2 (α = 0.76) and 6 months (α = 0.76).

#### 2.2.2. Feeding Styles, Milk Type, and Bottle Feeding

Information related to the use of breast milk vs. formula, use of bottle feeding, and other infant food-related behaviors and/or practices were collected using a mother-report via a modified version of the Infant Feeding Practices Questionnaire (IFPQ) [30]. A continuous variable of formula-feeding intensity was created from the food frequency portion of the IFPQ by calculating the proportion of all feeds using formula, ranging from 0 (breast milk only) to 1 (formula only). A continuous variable of bottle-feeding intensity was estimated by calculating the proportion of all feeds that were fed using a bottle (breastmilk or formula), with scores ranging from 0 (0% bottle feeding) to 1 (100% bottle feeding).

Participants’ feeding styles at the 2-month time point were assessed using the Infant Feeding Styles Questionnaire (IFSQ) [31]. The IFSQ has been validated with adequate reliability among infants, including low-income samples [26,31,32]. The IFSQ subscales include both behavioral (1—never to 5—always) and belief items (1—disagree to 5—agree) that reflect a specific feeding style. The following styles were assessed: (1) laisse-faire; (2) pressure; (3) restriction; (4) indulgent; (5) responsive. A higher score on each subscale reflects a greater use of the specific feeding style. Pressure (α = 0.80), restriction (α = 0.62), and indulgent (α = 0.91) feeding styles showed acceptable to good internal consistency. However, laisse-faire (α = 0.45) and responsive (α = 0.56) feeding styles were omitted due to their insufficient reliability.

#### 2.2.3. Sociodemographic, Anthropometric, and Other Characteristics

Sociodemographic information, such as maternal age, education attainment, race and ethnicity, income, marital status, household characteristics (i.e., other children in the home), living situation (i.e., living with a partner), and other information, was collected via Qualtrics prior to the prenatal lab visit. Maternal anthropometrics were collected via self-report (i.e., pre-pregnancy weight) and via lab measurements (i.e., weight, height) using standard procedures [33,34]. Maternal pre-pregnancy Body Mass Index (BMI) was calculated using self-reported pre-pregnancy weight and height that was measured during the prenatal visit (kg/m^2^). Infant birth-related information (i.e., birthweight) was collected via a self-report when researchers contacted participants by phone after birth (approximately 5 days post due date). Infant anthropometrics were assessed at the 2- and 6-month lab visits utilizing standard procedures that were developed by the World Health Organization, with details about the procedures and data collection described elsewhere [28]. Infants were weighed using a calibrated high-precision pediatric scale (Seca, Hamburg, Germany), and their recumbent length was measured using an infant measuring board (Perspective Enterprise, Portage, MI, USA). When possible, missing anthropometric information was retrieved with participants’ consent from their medical records.

### 2.3. Statistical Analyses

Preliminary analyses were conducted using SPSS Version 29 (IBM, Chicago, IL, USA). Bivariate correlations and *t*-tests were utilized to examine differences in 6-month FCR by selected determinants (e.g., race/ethnicity). Based on preliminary analyses and previous research, the following covariates were included in the final model: maternal age, race/ethnicity, income-to-needs ratio, infant sex, infant birthweight, and formula-feeding intensity. The interaction terms were specified in the final model, and final analyses were performed in Mplus 8.11 [35]. The proportion of missing data for the key study variables and covariates ranged from 0% to 20%. Missing data were handled via the full information maximum likelihood method [36].

In the final model, the moderating effects of each feeding style and bottle-feeding intensity on the association between 2-month and 6-month FCR were tested. First, product terms were generated by multiplying mean-centered variables of 2-month FCR, each feeding style (e.g., pressure), and bottle-feeding intensity. Second, product terms were specified as exogenous variables predicting 6-month FCR. Finally, any statistically significant interactions among 2-month FCR, specific feeding style, and bottle-feeding intensity were probed. To simplify the interpretation of complex 3-way interactions, a dichotomous variable of “high” vs. “low” bottle-feeding intensity was created by performing a median split to divide the sample in half based on the proportion of total feeds using a bottle [37]. Infants who received less than 50% of the feeds via bottle were categorized as “low”, and those who received 50% or more feeds via a bottle were classified as the “high” bottle-feeding group. The two-way interactions between the feeding style and 2-month FCR were examined within each group. Those that were significant were probed via simple slope analysis at +/−1*SD* of the moderator [38]. Covariates were included as exogenous variables to predict the outcome (i.e., 6-month FCR) while also correlating with predictors in all analyses. The unstandardized estimate (*b*), the standard error (*SE*) for unstandardized estimates, the 95% confidence intervals (CIs) for unstandardized estimates, the standardized estimate (β), and the *p*-values for standardized estimates were reported to identify interactive effects. The 95% CIs that do not include zero and the *p*-values less than 0.05 were considered statistically significant. The coefficient of determination (R^2^) was reported to indicate the proportion of variance in the dependent variable (i.e., 6-month FCR) that is explained by the model.

## 3. Results

A total of 299 enrolled pregnant women (aged 18 to 47 years, *M* = 29.71, *SD* = 5.48) were included in the final model. Approximately 53% women self-identified as non-Hispanic White, 29% as non-Hispanic Black, and 19% as other. The median annual household income was $62,500, and the mean value of the household income-to-needs ratio was 3.49 (*SD* = 2.96). When infants were 2 months old, 51% of mothers exclusively used breast milk, 22% exclusively used formula, and 27% used a mix of breast milk and formula to feed their infant (i.e., formula-feeding intensity continuous proportion score, *M* = 0.33, *SD* = 0.41). Out of 249 infants whose mothers reported their bottle-feeding intensity at 2 months, 124 (49.8%) were categorized into the “low” bottle-feeding group and 125 (50.2%) were categorized into the “high” bottle-feeding group. The characteristics of the infants are reported in Table 1. Zero-order bivariate correlations among study variables are presented in Table 2. FCR at 2 months, pressure and restriction feeding styles, and formula-feeding intensity were positively associated with 6-month FCR, ranging from small to moderate in magnitude [39].

**Figure 1:**
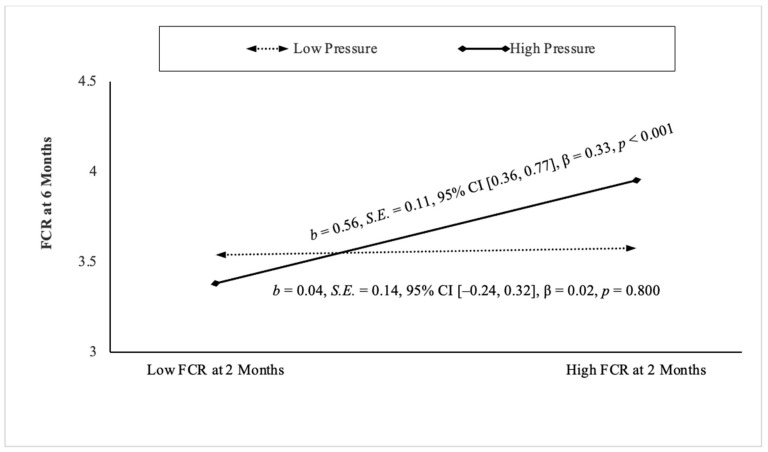
The association between food cue reactivity (FCR) at 2 months and 6 months at low and high levels of pressure feeding among infants in the “high” bottle-feeding group (*n* = 125).

Pressure was the only feeding style that interacted with bottle-feeding intensity and 2-month FCR in predicting 6-month FCR (Table 3). Specifically, pressure moderated the association between 2-month and 6-month FCR among infants in the “high bottle-feeding” group (*b* = 0.50, *S.E.* = 0.16, 95% CI [0.19, 0.80], β = 0.29, *p* = 0.001), but not in the “low bottle-feeding” group (*b* = −0.26, *S.E.* = 0.22, 95% CI [−0.69, 0.18], β = −0.12, *p* = 0.254). For infants in the “high bottle-feeding” group (Figure 1), greater 2-month FCR predicted higher 6-month FCR at higher levels of maternal pressure feeding only, not at lower levels of pressure. No other significant three-way or two-way interactions were detected in the model. The proposed model explained 28% (R^2^ = 0.28) of the variance in 6-month FCR, indicating a large effect size [39].

## 4. Discussion

Our study identified a key interaction among early FCR, pressure to eat, and bottle feeding that is associated with increased food approach traits later in infancy. Among primarily bottle-fed infants, maternal pressure during feeding exacerbated the positive association between infant FCR at 2 months and FCR at 6 months of age. The other feeding styles tested in our model, restrictive and indulgent, did not interact with early FCR nor bottle feeding to influence infants’ FCR at 6 months. Several studies have examined FCR among children [11,13,16], but our study contributes new knowledge on FCR and its interactions with feeding styles and feeding mode among infants. Our findings can be used to make more specific recommendations for optimal infant feeding and/or to inform the design of future feeding-related interventions for parents of young infants.

Breastmilk is the gold standard for feeding infants [40]. However, only 26% of infants in the U.S. are being exclusively breastfed in their first 6 months of life [41]. As a result, most parents use a bottle for some proportion of the infants’ feeds, especially as more mothers have been utilizing the “pumping and feeding” method with breastmilk in recent years [42]. Thus, rather than the milk type, we tested feeding mode in our study as a key moderator of the interactions between early and later FCR during infancy. In our “high” bottle-fed group, infants with higher food approach traits, whose mothers used more pressure during feeding early in infancy, had a greater FCR at 6 months. Maternal pressure has long been considered an obesogenic feeding practice, since it is believed to override children’s natural food cues and thus disrupt self-regulation of food intake [20,21]. However, most of the existing research on maternal pressure has, so far, come from preschool-aged or older children, with little knowledge on infants [21,43,44,45]. Furthermore, the nature of the associations between maternal pressure during infancy and weight outcomes remains unclear due to a scarcity of studies that would clarify the direction of these associations [46]. To date, maternal pressure has been found to be both inversely correlated with weight (i.e., some mothers use more pressure when they perceive their child to be underweight), or uncorrelated with weight outcomes across studies [46,47]. These inconsistent findings may be due to the cross-sectional nature of most studies, where the direction of the associations cannot be determined [43,44,48].

Currently, there is very limited knowledge related to maternal pressure in relation to infant appetite [44,49]. A recent cross-lagged model analysis by Eagleton et al. [49] found that maternal feeding pressure was associated with significant increases in infant food responsiveness. Additionally, food to soothe, which is also a pressuring feeding practice, was found to have similar, that is increasing, effects on infant food responsiveness. The fact that feeding mode emerged as a significant moderator of the interactions between early FCR and pressure in our sample is not surprising. Associations have been found between bottle feeding (regardless of milk type) and a higher risk of obesity, including a greater rapid infant weight gain [27,50,51]. Many existing studies, however, confound the effects of milk type and feeding mode on infant outcomes [22]. Here, we specifically examined the moderating effect of bottle-feeding intensity by controlling for the milk type in our analyses.

In our sample, early FCR interacted with greater maternal pressure to increase infants’ FCR later, but only among infants who received most of their feeds via a bottle. Previous research offers possible explanations for our findings. First, mothers who bottle-feed report a greater tendency to encourage their infant to finish/empty the bottle compared to mothers who feed at the breast [21]. Second, pressure is generally easier to apply when using a bottle than when feeding at the breast because mothers have more control over the feeding and can see how much milk is left during a feed [52]. Finally, mothers might choose to use a larger bottle than is appropriate for the infant’s age, which may promote offering larger milk volumes, thus leading to infant overfeeding and potentially higher obesity risk later in life [23,27,52,53]. Our study contributes to the limited literature on appetite traits among infants, demonstrating that maternal pressure early in life, especially among infants who are primarily bottle-fed, promotes greater increases in food approach traits among infants who show higher interest in food early on, thus potentially further increasing their obesity risk.

Our findings highlight the need to educate parents not only about “what” but also “how” to feed their infants and to develop effective and practical guidelines for parents who use mixed feeding (i.e., both at breast and bottle feeding) or for those who only bottle feed [22]. In recent years, a few innovative interventions targeting bottle feeding practices have been developed [54,55]. Findings suggest that simple tips, such as using opaque and/or weighted bottles and paced bottle feeding during infant feeding, are associated with increased maternal sensitivity, slowed rate of feeding, and extended meal duration [54,55]. Thus, these tips may work as counter-strategies when infants are bottle-fed and may prevent excessive pressure and/or overfeeding during infancy [52,54,55].

Our non-significant findings related to restrictive and indulgent feeding styles are not surprising, as feeding behaviors are developmentally specific. For instance, the use of restriction or indulgent feeding likely matters more when children are older, when exposed to a variety of foods, as opposed to young infants who only consume either breast milk or formula. In older samples, children whose mothers reported high levels of restriction had a significantly greater appetite and consumed substantially greater amounts of energy during a lab task than children whose mothers reported using less restriction [56,57]. Similarly, an indulgent feeding style has been associated with greater obesity risk in previous studies with older children [45]. However, our study shows that during infancy, restriction or indulgent feeding do not interact with early appetite or feeding mode to influence infants’ later FCR.

Our study has several strengths as well as limitations. First, FCR was operationalized as a score of three food approach traits rather than a single food approach trait, such as food responsiveness or enjoyment of food only, as used in previous research. Second, most of the infant anthropometrics (except birth data) were assessed by our trained research staff as opposed to relying on maternal reports. Third, our sample was socio-economically and racially/ethnically diverse, with a wide range of maternal education as well as income. Finally, to our knowledge, this is the first study to examine interactions between early FCR, feeding styles, and feeding mode on later FCR among infants. Our study also has several limitations. First, a certain level of social desirability and/or bias should be assumed in the maternal report of feeding styles as well as infant appetite traits. Second, given poor reliability, we were unable to examine whether laisse-faire and responsive feeding styles at 2 months also moderate FCR over time. However, we were able to test key feeding styles that have been implicated in increased obesity risk and/or food approach traits in previous studies [19,26]. Third, our study was conducted in an urban area in the southeast of the U.S., and thus our findings cannot be generalized to samples of mother–infant dyads, such as those living in rural areas and low-income households. Finally, our findings should be replicated in larger samples that extend the analyses to determine and/or confirm the direction of the associations between FCR in early infancy, maternal food-related practices, and FCR through the first 2 years of life.

## 5. Conclusions

To date, very few studies have examined how early appetitive traits interact with maternal feeding-related behaviors and FCR over time during infancy. The current study fills an important gap by identifying specific interactions between early food approach traits and feeding styles and bottle feeding that promote a greater FCR during infancy. Our study shows that pressure, one of the controlling feeding styles, uniquely interacts with infant early FCR and bottle feeding to influence FCR a few months later. Because infant rapid weight gain is a robust risk factor for future obesity [58], prevention of large FCR increases early in life might be an important target for future interventions. However, further research is warranted to investigate these associations in more depth, using objective measures of FCR and feeding styles in diverse and large samples, and to establish the direction of these associations from early infancy to preschool age via longitudinal studies.

Our findings also have implications for practice. Many new parents rarely receive education and support for infant feeding beyond the breastfeeding recommendations [59]. Given our findings and the negative feeding- and weight-related infant outcomes linked to pressure and bottle use during feeding in previous studies, specific education on minimizing maternal pressure and providing tips on how to optimize infant bottle-feeding should be provided to new parents. Given the limited resources that are currently available to parents, especially those who want and/or need to bottle-feed frequently, such guidance and support is timely and it might potentially reduce the risk of rapid weight gain in some infants, similar to those in our sample, in the first year of life [6,60,61].

## Figures and Tables

**Table 1:** Infant Characteristics and descriptives for key study variables.

Characteristics/Variables	*N*	*M* (*SD*)/%	Range
Infant birth weight (kg)	288	3.36 (0.49)	1.56–4.76
Infant length (cm)	278	50.46 (2.59)	41.28–57.15
Infant sex ^1^	289	51.2 ^2^	–
Infant age at 2 m visit (months)	240	2.27 (0.50)	1.71–4.47
FCR 2 m	258	3.72 (0.51)	2.31–4.83
Pressure 2 m	255	2.13 (0.53)	1.00–3.97
Restriction 2 m	255	3.05 (0.64)	1.45–4.70
Indulgent 2 m	253	1.52 (0.51)	1.00–3.70
Bottle-feeding intensity 2 m	249	0.51 (0.42)	0–1.00
FCR 6 m	238	3.48 (0.51)	2.03–4.94

Note: *N* = valid sample size; *M* (*SD*) = mean and standard deviation; FCR = food cue reactivity; kg = kilogram; cm = centimeter; m = months. ^1^ Coded as 1 = male and 2 = female; ^2^ Percentage of males, based on its valid sample size (289).

**Table 2:** Zero-order bivariate intercorrelations among key study variables and covariates.

Variable	1	2	3	4	5	6	7	8	9	10	11	12
1. FCR 2 m	–											
2. Pressure 2 m	0.18 **	–										
3. Restriction 2 m	0.18 **	0.31 **	–									
4. Indulgent 2 m	−0.01	0.44 **	−0.06	–								
5. Bottle feeding 2 m ^1^	−0.03	0.27 **	0.33 **	0.22 **	–							
6. FCR 6 m	0.48 **	0.23 **	0.22 **	0.08	0.05	–						
Covariates												
7. Infant sex ^2^	−0.14 *	−0.11	−0.07	−0.07	−0.13 *	−0.10	–					
8. Infant birth weight (kg)	0.06	−0.09	−0.04	−0.11	−0.11	0.05	−0.08	–				
9. Formula-feeding 2 m ^3^	0.04	0.34 **	0.29 **	0.27 **	0.76 **	0.15 *	−0.04	−0.15 *	–			
10. Maternal age	−0.12	−0.14 *	−0.11	−0.20 **	−0.08	−0.15 *	−0.07	0.03	−0.18 **	–		
11. Maternal race/ethnicity ^4^	0.15 *	0.34 **	0.29 **	0.22 **	0.23 **	0.16 *	0.02	−0.27 **	0.27 **	−0.18 **	–	
12. Income-to-needs ratio	−0.12	−0.21 **	−0.16 *	−0.17 **	−0.10	−0.14 *	−0.05	0.07	−0.26 **	0.29 **	−0.42 **	–

Note: FCR = food cue reactivity; kg = kilogram; m = months; * *p* < 0.05, ** *p* < 0.01. ^1^ Bottle-feeding intensity (proportion of total feeds from a bottle); ^2^ Coded as 1 = male and 2 = female; ^3^ Formula-feeding intensity (proportion of total feeds using an infant formula); ^4^ calculated as a dichotomous variable; 0 = Non-Hispanic White; 1 = all other.

**Table 3:** Regression results for the three-way interaction model.

Outcome: FCR 6 Months	b (SE)	*p*	β
Main Effects			
FCR 2 months	0.42 (0.07)	**<0.001**	0.42
Pressure 2 months	0.06 (0.07)	0.419	0.06
Restriction 2 months	0.08 (0.06)	0.182	0.09
Indulgent 2 months	0.02 (0.07)	0.712	0.03
Bottle-feeding intensity 2 months	−0.11 (0.12)	0.372	−0.09
2-way interaction terms			
Pressure 2 m X FCR 2 months	0.11 (0.14)	0.459	0.06
Restriction 2 m X FCR 2 months	−0.05 (0.09)	0.569	−0.03
Indulgent 2 m X FCR 2 months	0.06 (0.16)	0.714	0.03
Bottle-feeding intensity 2 m X FCR 2 months	−0.25 (0.15)	0.101	−0.11
3-way interaction terms			
Pressure 2 m X Bottle-feeding intensity 2 m X FCR at 2 months	0.79 (0.37)	**0.034**	0.17
Restriction 2 m X Bottle-feeding intensity 2 m X FCR at 2 months	−0.43 (0.22)	0.053	−0.12
Indulgent 2 m X Bottle-feeding intensity 2 m X FCR at 2 months	−0.52 (0.37)	0.158	−0.12
Covariates			
Infant sex ^1^	−0.05 (0.06)	0.406	−0.05
Infant birth weight (kg)	0.05 (0.07)	0.468	0.05
Formula-feeding intensity	0.13 (0.12)	0.296	0.10
Maternal age	−0.01 (0.01)	0.322	−0.05
Maternal race/ethnicity ^2^	0.03 (0.07)	0.656	0.03
Income-to-needs ratio	−0.00 (0.01)	0.717	−0.03

Note: Bold font indicates significant effects at *p* < 0.05. FCR = food cue reactivity; kg = kilogram; ^1^ Coded as 1 = male; 2 = female; ^2^ calculated as a dichotomous variable; 0 = Non-Hispanic White; 1 = all other.

## Data Availability

The original data presented in the study are openly available in Harvard Dataverse at https://dataverse.harvard.edu/dataset.xhtml?persistentId=doi:10.7910/DVN/0UVL7L (accessed on 16 November 2025).
